# On the Treatment of Contractions and Anchylosis of the Knee and Hip Joint by Forced Rupture

**Published:** 1854-07

**Authors:** 


### ECLECTIC DEPARTMENT,

### AND SPIRIT OF THE MEDICAL PERIODICAL PRESS.

---

*On the Treatment of Contractions and Anchylosis of the Knee and Hip Joint by Forced Rupture.* By P. Frank, M. D., of Manchester.

In our dissertation on contractions and anchyloses, we have expressed our full approbation of the opinion first promulgated by Bonnet, viz., that it is not only justifiable, but even strictly indicated, to stretch a contracted joint, although inflammatory symptoms may still exist. The theoretical presumptions by which this practice is vindicated are too well known to the profession to need any further discussion. It is a practice which has been crowned with the most eminent success, as all those conversant with the works of Bonnet are aware of. A most brilliant case of this kind, which occurred in the practice of Mr. Langenbeck, is subsequently related, which will testify more forcibly in favor of the proposition we advance than the most eloquent arguments. This practice is, of course, contra-indicated if the organism of the patient is in an advanced state of debility, where a dangerous and local reaction might be apprehended. According to the experience of Mr. Langenbeck, a still existing chronic-arthritic process can alone be regarded as an absolute contra-indication. If this process has terminated, contractions of the joint can be successfully treated by forced extension. The deep alterations known to be produced on the articular surfaces by the chronic-arthritic processes do not permit us to expect the restoration of the normal functions of the joint.

It will be no matter of surprise, that forced extension being practiced under the precautions enumerated, we have no account to give of serious accidents having supervened during the operation. The skin was never ruptured. Only in one case superficial excoriations were observed in the popliteal fossa. We never saw the popliteal artery ruptured as it was by the machine of Louvrier, nor was ever one of the large nerves lacerated. Only once, after forced extension of an anchylosis at an acute angle, anæsthesia supervened in that part of the skin supplied by the saphenus major nerve. In a short time, however, the normal sensibility returned. Nothing more serious than a distension of the nerve can, therefore, be surmised. Where a subluxation of tibia backward existed before the operation, it of course became more evident, after extension had been performed. Only in two cases, one of which occurred in the Royal Surgical Clinic, the other in private practice of Mr. Langenbeck, the luxation was rendered perfect. In both of these cases, it was impossible to detach the anchylosed patella from the external condyle of the femur. All the injuries produced by the operation being subcutaneous, the reaction, after a proceeding apparently so dangerous, never attained a serious character. Of constitutional symptoms nothing but a slight febrile irritation is on record.

As far as local action is concerned, in most cases nothing but a superficial reddening was observed. In some cases, a slight inflammatory infiltration of the parts surrounding the joint took place; in others, small sugilations were noticed. A suppurative phlegmon was never remarked. These symptoms quickly yielded, in all cases, to the employment of cold applications. Violent pains in the stretched muscles always followed the operation. In several cases pains increasing on pressure were complained of on the anterior surface of the epiphysis of the tibia. We do not hesitate to ascribe these to violent contusions to which the part is exposed in attempting to detach the anchylosed patella. They were most marked in those cases where this detachment was attended with the greatest difficulties.—*Med. Times & Gazette.*

